# A Multi-Integrated Approach to Tinnitus Phenotype-Guided Otolaryngological, Dental, and Physiotherapeutic Care

**DOI:** 10.3390/life16101588

**Published:** 2026-09-23

**Authors:** Alessandro Carollo, Daniele Puzzilli, Pasquale Longo, Alessandro Conforti, Lino Di Rienzo Businco, Bruno Brandimarte

**Affiliations:** 1 International Osteopathic Institute, 00135 Rome, Italy; 2 Dentistry, Institute of Sports Medicine and Science (CONI), 00197 Rome, Italy; 3 Clinical Research Unit, Fondazione Integria ETS, 00197 Rome, Italy; 4 Rheumatology Unit, ASL Roma 4, 00053 Civitavecchia, Italy; 5 Department of Otorhinolaryngology and Audiology, San Giovanni Battista Hospital Military Sovereign Order of Malta, 00148 Rome, Italy; 6 Ultrastructural Biophysics Research Institute and Healthcare Technologies (UBRI), 00197 Rome, Italy; 7 Longevìa Clinic, 00187 Rome, Italy

**Keywords:** tinnitus, multidisciplinary care, integrated care, audiology, temporomandibular disorders, somatosensory tinnitus, physiotherapy, rehabilitation, personalized care

## Abstract

**Background:** Tinnitus is a heterogeneous percept that becomes a disorder when it is accompanied by clinically meaningful distress or functional impairment. Hearing loss is a major contributor, while temporomandibular, cervical, sleep, psychological, and contextual factors can shape symptom expression. This heterogeneity supports coordinated assessment, but it does not by itself prove that an integrated care pathway improves outcomes. **Objective:** To narratively synthesize evidence relevant to phenotype-guided tinnitus care and to propose a clinical–organizational framework for coordinating otolaryngological and audiological care, dental and temporomandibular management, physiotherapeutic rehabilitation, and complementary behavioral and sleep interventions. **Methods:** A structured narrative review was developed with reference to SANRA. PubMed/MEDLINE, the Cochrane Library, major guideline repositories, and targeted citation searches were examined for English-language evidence from January 2014 to 12 August 2026, with seminal earlier publications retained. Guidelines, systematic reviews, randomized trials, and clinically informative observational studies were prioritized. Owing to substantial heterogeneity, findings were synthesized thematically rather than pooled. **Results:** The evidence separates into three levels. Guideline-supported or relatively strong evidence underpins diagnostic assessment, education, cognitive behavioral therapy, and hearing rehabilitation when indicated. Temporomandibular and cervical interventions have more limited, phenotype-specific evidence from small and heterogeneous studies. No comparative study has validated the proposed integrated framework as a complete treatment package. We therefore present coordination, phenotype-gated referral, shared outcomes, and explicit adaptation or stop rules as a hypothesis-generating organizational model. **Conclusions:** The proposed framework should be regarded as a hypothesis-generating organizational model rather than a validated treatment package. Its individual components retain their own levels of evidence, and prospective studies should compare the coordinated framework with usual or standard care using both clinical and health-economic outcomes.

## 1. Introduction

Tinnitus is the perception of sound without a corresponding external acoustic source. An international proposal usefully distinguishes the percept itself from tinnitus disorder, in which the percept is accompanied by clinically relevant emotional, cognitive, behavioral, or functional consequences [1]. This distinction prevents two common errors: treating every tinnitus percept as a disease requiring intervention, and reducing severe tinnitus disorder to a simple psychoacoustic phenomenon. Global estimates vary with case definition and sampling, but tinnitus is common, while only a subset of affected people experience sustained disability or seek specialist care [2,3].

The clinical problem is defined less by prevalence than by heterogeneity. Tinnitus may follow or accompany sensorineural hearing loss, noise exposure, otologic disease, medication exposure, head or neck trauma, temporomandibular dysfunction, or other conditions; in many patients, no single cause explains the ongoing burden. Its salience can fluctuate with external sound, stress, sleep, attention, jaw activity, neck movement, and context [4]. Accordingly, patients with similar descriptions of pitch or loudness may have very different hearing profiles, comorbidities, modulating factors, coping resources, and treatment priorities [4,5,6].

Contemporary guidance supports multidisciplinary assessment, while practice remains variable across services and countries [7,8]. Meijers et al. systematically compared chronic tinnitus guidelines and showed both areas of agreement and important variation in treatment recommendations [9]. The present narrative review does not aim to replace that systematic guideline review. Instead, it asks how established and phenotype-specific components can be coordinated without indiscriminate treatment bundling. Referral pathways may be serial and fragmented, but the opposite extreme is also problematic: more treatment is not necessarily more integrated care.

Three related terms should be separated. Multimodal care combines more than one therapeutic modality, while multidisciplinary care involves more than one profession. In this review, multi-integrated care refers to an operational layer in which participating disciplines share a formulation, agree on sequencing, use common outcomes, identify a coordinator, and reconvene to continue, modify, de-escalate, or stop components. The term is used as a practical organizational concept, not as established nomenclature. We do not claim that multidisciplinary or coordinated tinnitus care is new; the purpose is to make its decision rules explicit enough to be described, audited, and prospectively tested.

This review focuses on a clinically coherent axis comprising otolaryngology/audiology, dentistry and temporomandibular care, and physiotherapy for cervical or craniomandibular dysfunction. Behavioral treatment, sleep care, communication rehabilitation, and participation-oriented support are retained as complementary domains because they address major drivers of tinnitus burden and, for cognitive behavioral therapy (CBT), have a stronger evidence base than many percept-directed interventions. Emerging technologies are considered only as future extensions of this clinical architecture.

The aims are to: (1) explain why tinnitus heterogeneity supports phenotype-guided assessment; (2) review the assessment and management roles of otolaryngology and audiology, dental and temporomandibular care, and physiotherapy; (3) propose a hypothesis-generating organizational framework for coordinating these disciplines; (4) identify safeguards against overdiagnosis and overtreatment; and (5) define research priorities for phenotyping, prospective pathway evaluation, shared outcomes, and responsible technological development.

### Key Messages

1. Tinnitus heterogeneity requires phenotype-guided assessment rather than indiscriminate treatment bundling.
2. ENT and audiology provide the safety, diagnostic, hearing, and communication foundation of care.
3. Eustachian tube dysfunction is relevant as an otologic differential diagnosis when symptoms and objective middle-ear findings are compatible. Aural fullness or a high symptom-questionnaire score alone should not be interpreted as evidence that Eustachian tube dysfunction is causing tinnitus.
4. Dental and temporomandibular management is relevant only when history and examination support a credible TMD-associated orofacial phenotype; the evidence for tinnitus improvement remains phenotype-specific and limited.
5. Cervical or craniomandibular physiotherapy may be considered when examination findings and tinnitus behavior co-vary, but the available trials are small and heterogeneous. No specific manual, manipulative, mobilization, soft-tissue, or active technique has established superiority.
6. Coordination, shared outcomes, and planned reassessment define the proposed multi-integrated framework. The framework itself is not yet a validated treatment protocol.
7. Behavioral, sleep, digital, and emerging technological approaches should complement the clinical pathway without displacing evidence-based assessment or overstating efficacy.

## 2. Methods

### 2.1. Review Design

A structured narrative review was selected because the review question crosses mechanistic, diagnostic, rehabilitative, behavioral, and service-delivery literatures that are not amenable to a single pooled effect estimate. The manuscript was developed with reference to the Scale for the Assessment of Narrative Review Articles (SANRA), emphasizing a justified scope, explicit aims, a transparent search description, appropriate referencing, scientific reasoning, and evidence-calibrated presentation [10]. Narrative synthesis was used as an interpretive method rather than as a substitute for critical appraisal [11].

### 2.2. Information Sources and Search Strategy

PubMed/MEDLINE and the Cochrane Library were searched for English-language literature published from 1 January 2014 through 12 August 2026. Seminal earlier publications were retained when they defined diagnostic criteria, outcome instruments, foundational mechanisms, or enduring clinical recommendations. The searches combined tinnitus with terms covering hearing loss, audiology, hearing aids, cochlear implantation, sound therapy, somatosensory or somatic tinnitus, temporomandibular disorders, bruxism, orofacial pain, cervical dysfunction, physiotherapy or physical therapy, rehabilitation, cognitive behavioral therapy, sleep or insomnia, nasal obstruction, Eustachian tube dysfunction or stenosis, middle-ear ventilation, hypoventilation or hypoxia, multidisciplinary or integrated care, digital monitoring, ecological momentary assessment, and neuromodulation.

Major clinical guidance was identified from the National Institute for Health and Care Excellence (NICE), the US Department of Veterans Affairs/Department of Defense (VA/DoD), the American Academy of Otolaryngology–Head and Neck Surgery Foundation (AAO-HNSF), the German S3 program, and the European multidisciplinary tinnitus guideline [7,12,13,14,15]. The systematic guideline review by Meijers et al. was used to contextualize agreement and disagreement across published recommendations [9]. The search strategy was developed by the author team. Targeted backward and forward citation searching was used to locate pivotal trials and recent reviews. Potentially relevant sources were assessed against the prespecified scope, evidence hierarchy, population, and clinical applicability. Duplicate reports and sources outside the adult subjective non-pulsatile tinnitus scope were excluded. The final search was run on 12 August 2026. Study selection was not performed independently in duplicate, and no formal saturation threshold was used. Main search strings and further selection details are provided in Supplementary Tables S1 and S2.

### 2.3. Selection and Interpretation

Priority was given to clinical practice guidelines, systematic reviews, randomized trials, and prospective comparative studies. Observational studies were used for prevalence, association, feasibility, and phenotype description, not as proof of treatment efficacy. Sources were selected for direct relevance to adult subjective non-pulsatile tinnitus or tinnitus disorder; publications on pulsatile tinnitus, objective somatosounds, acute otologic emergencies, and pediatric populations were retained only where necessary for diagnostic triage. Patent documents, promotional materials, and unverified efficacy claims were not treated as clinical evidence.

No meta-analysis was performed. Strength of language was matched to study design, consistency, directness, and clinical applicability. To make the evidence hierarchy explicit, findings were organized into three positions: (1) interventions supported by established tinnitus guidelines or relatively strong evidence, including core assessment, education, cognitive behavioral therapy, and hearing rehabilitation when indicated; (2) phenotype-specific interventions supported by limited or emerging evidence, including TMD-directed treatment and cervical physiotherapy; and (3) the proposed coordination framework itself, which is treated as a hypothesis-generating organizational model rather than a validated treatment package. Associations between tinnitus and temporomandibular or cervical findings were not interpreted as proof of causation. The absence of a formal systematic-review flow diagram and duplicate risk-of-bias scoring is acknowledged as a limitation of the narrative design.

## 3. Why a Multi-Integrated Approach Is Biologically and Clinically Plausible

### 3.1. Auditory Deafferentation and Distributed Network Adaptation

Hearing loss is the most consistently recognized tinnitus risk factor, but peripheral damage does not map one-to-one onto the percept. Reduced cochlear or auditory-nerve input can be followed by compensatory changes in central gain, spontaneous activity, synchrony, and network weighting. Attention, salience, limbic, and autonomic systems influence whether the percept becomes intrusive or distressing [3,4,5,6]. These mechanisms support audiological evaluation and hearing rehabilitation, but they do not imply that every patient has a single auditory lesion or that normalization of one psychoacoustic feature will resolve tinnitus disorder.

### 3.2. Auditory–Somatosensory Interaction

Somatosensory pathways from the trigeminal system and upper cervical region converge with auditory processing at brainstem and higher levels. Experimental models describe plastic changes in auditory–somatosensory integration after auditory deafferentation, including altered weighting of somatosensory input to the cochlear nucleus [16]. Clinically, some patients can change tinnitus loudness or pitch through jaw clenching, neck movement, pressure on muscles, or other maneuvers. Such modulation is common enough that it cannot by itself establish a somatosensory diagnosis. Consensus criteria place greater weight on temporal co-variation between tinnitus and jaw or neck symptoms, simultaneous onset or aggravation, relevant trauma, and reproducible modulation in the context of a compatible examination [17,18,19].

Systematic review evidence supports an association between tinnitus and temporomandibular disorders (TMD), and a weaker association with cervical disorders, but the included studies are heterogeneous and vulnerable to selection and measurement bias [20]. The appropriate inference is therefore conditional: jaw or cervical dysfunction may be a clinically relevant contributor in a subgroup, not a universal explanation for tinnitus.

### 3.3. Distress, Sleep, Attention, and Participation

Tinnitus severity is only partly related to perceived loudness. Threat appraisal, selective attention, avoidance, low self-efficacy, anxiety, depression, hyperacusis, insomnia, and communication difficulty can sustain disability. These domains are not secondary in the sense of being unimportant or imaginary; they are mechanisms through which a persistent percept acquires personal and functional significance. A multi-integrated model must therefore combine phenotype-specific investigation with an explicit account of distress, sleep, social participation, and patient goals.

### 3.4. From Multiple Disciplines to Genuine Integration

Biological plausibility does not validate a treatment package. Coordination may still have practical value if it improves selection, reduces contradictory advice or duplicated care, and supports timely adaptation. An audiologist, dentist, and physiotherapist may each identify clinically relevant targets, but those findings should be separated into confirmed diagnoses, plausible contributors, and uncertainty. Whether this organizational approach improves patient outcomes or efficiency remains a testable hypothesis.

## 4. Phenotype-Guided Assessment Before Treatment

### 4.1. Safety, Red Flags, and Otologic Differential Diagnosis

Assessment begins with the characteristics of the sound, time course, laterality, pulsatility, associated hearing or vestibular symptoms, medication and noise history, neurologic symptoms, trauma, and the degree of distress. Otoscopy and audiological assessment are foundational. Sudden hearing loss, acute neurologic deficit, severe acute vestibular symptoms, pulsatile tinnitus, objective somatosound, or a mental health crisis requires an urgent or condition-specific pathway. Persistent unilateral tinnitus, asymmetric hearing loss, or focal neurologic findings may justify specialist evaluation and targeted imaging. Routine imaging for symmetrical non-pulsatile tinnitus without localizing findings is not supported [12,13,14,15].

Treatable causes should be managed according to the underlying condition, for example cerumen impaction, middle-ear disease, sudden sensorineural hearing loss, otosclerosis, Ménière disease, vestibular schwannoma, or a vascular lesion. This is not the same as claiming that treatment of every associated condition will eliminate chronic tinnitus. The initial consultation should explain both what has been excluded and what remains uncertain.

Eustachian tube dysfunction (ETD) belongs in the otologic differential when aural fullness, popping, fluctuating conductive hearing symptoms, tympanic-membrane abnormalities, or abnormal tympanometry suggest middle-ear pressure dysfunction. Tinnitus can accompany ETD, but symptoms alone do not establish ETD as the cause of tinnitus. Recent data show that ETDQ-7 scores have low sensitivity and specificity for objectively confirmed ETD and, in patients with tinnitus, may reflect general tinnitus-related discomfort rather than tubal dysfunction [21,22]. When ETD is suspected, diagnosis should therefore rest on clinical examination and objective middle-ear testing rather than questionnaire score alone.

### 4.2. Audiological and Communication Profile

Pure-tone audiometry is the minimum audiological baseline for most patients seeking care, supplemented as indicated by speech testing, tympanometry, uncomfortable loudness levels, or other tests. The assessment should determine whether hearing loss affects communication, listening effort, work, safety, or participation. Tinnitus pitch and loudness matching can characterize the percept but are not reliable proxies for distress and should not be used as the sole treatment outcome. Hyperacusis and sound avoidance require careful differentiation because overprotection can increase disability, while indiscriminate sound exposure can be aversive.

### 4.3. Dental and Temporomandibular Phenotype

A dental or orofacial referral is most credible when tinnitus onset or fluctuation tracks jaw pain, jaw function, parafunctional loading, or a defined TMD; when jaw maneuvers reproducibly modulate tinnitus; or when the patient has clinically meaningful masticatory symptoms. The Diagnostic Criteria for Temporomandibular Disorders (DC/TMD) provide a standardized physical and psychosocial assessment framework [23]. Relevant features include familiar pain on palpation or movement, arthralgia, myalgia, restricted or painful mandibular movement, joint symptoms, and the functional and psychosocial impact of TMD.

Bruxism may be relevant when it contributes to muscle or joint loading, but self-reported clenching or grinding should not be converted automatically into a causal tinnitus diagnosis. Occlusion, minor imaging abnormalities, and joint sounds are also common in people without tinnitus. The dental formulation should state the TMD diagnosis and treatment goal independently of the expectation that tinnitus will change.

### 4.4. Cervical and Physiotherapeutic Phenotype

Physiotherapeutic assessment is most useful when tinnitus coexists with neck pain, headache, reduced or painful movement, relevant trauma, sustained load intolerance, or reproducible co-variation between cervical symptoms and tinnitus. The examination should focus on neurologic and vascular warning signs, movement and load tolerance, strength and endurance, task-specific aggravation, and patient behavior rather than an aesthetic concept of ideal posture. Palpation-induced modulation may contribute to the formulation but is not a stand-alone diagnostic test.

### 4.5. Burden, Sleep, Mental Health, and Patient Goals

Validated patient-reported outcome measures should quantify impact rather than simply record presence. The Tinnitus Functional Index (TFI) was designed to measure severity and treatment-related change, while the Tinnitus Handicap Inventory (THI) remains widely used [24,25]. Core outcome work emphasizes tinnitus intrusiveness or distress, ability to ignore, sleep, concentration, emotional impact, and quality of life, with the selected domains matched to the intervention [26]. Scores are not interchangeable, and questionnaire content differs; consistent use of the same instrument across follow-up is more important than collecting many partially overlapping scales [27].

Clinical review should also screen for insomnia, anxiety, depression, suicidal thinking, hyperacusis, hearing-related participation restriction, and the patient’s own priorities. A concise goal statement—for example, sleeping through the night, returning to meetings, reducing jaw pain, or tolerating desk work—anchors shared decision-making and helps prevent indefinite treatment in the absence of meaningful change. The assessment domains, candidate phenotypes, primary roles, and shared outcomes are summarized in Table 1.

## 5. Evidence-Informed Management by Clinical Domain

### 5.1. Otolaryngological and Audiological Management

Condition-specific ENT treatment is appropriate when examination identifies a treatable otologic or middle-ear disorder. For objectively confirmed ETD, management should follow standard ENT indications and target the underlying condition rather than tinnitus itself. Evidence does not support nasal obstruction or suspected ETD as general tinnitus-treatment targets, and tinnitus-specific benefit from ETD-directed procedures has not been established [21,22]. Respiratory hypoventilation, sleep apnea, or clinically significant hypoxemia should be evaluated on their own medical indications and are not presented here as components of tinnitus treatment.

#### 5.1.1. Education, Explanation, and Shared Decision-Making

Education is a treatment component, not merely a prelude to treatment. It should provide a clear account of the assessment, correct catastrophic interpretations, explain the distinction between tinnitus percept and tinnitus disorder, and set realistic goals. Patients should protect against genuinely hazardous noise without routine overprotection from ordinary sound. Counseling and education are consistently supported across major guidelines and their systematic comparison [7,9,12,13,14,15].

#### 5.1.2. Hearing Rehabilitation

Hearing aids should be offered when hearing loss affects communication, following standard candidacy, fitting, verification, and follow-up principles. Their primary indication is hearing and communication rather than a promise to eliminate tinnitus. Amplification may reduce listening effort, increase access to environmental sound, and reduce the contrast between tinnitus and silence. Hearing aids improve hearing-related quality of life in adults with mild-to-moderate hearing loss [28]. Tinnitus-specific studies frequently report improvement, but designs and outcome measures are heterogeneous; verified amplification appears more favorable than inadequately matched amplification [29,30].

Cochlear implantation can reduce tinnitus in many people who meet hearing-loss candidacy, including selected patients with single-sided deafness, but tinnitus outcomes vary and new or worsened tinnitus can occur. Implantation should be governed by established auditory indications and informed consent, not by tinnitus alone [31]. Communication strategies, assistive listening technology, and workplace adaptation should accompany amplification when participation is limited.

#### 5.1.3. Sound Enrichment

Environmental sound, bedside sound, apps, hearing-aid sound generators, or individualized sound can provide distraction, reduce contrast, or support sleep. Evidence does not establish a universally superior sound type or device. A Cochrane review found low-certainty evidence and no clear superiority among hearing aids, sound generators, and combination devices [32]. Sound should therefore be framed as an optional self-management tool linked to a specific goal. It should be comfortable, should not obscure communication or safety signals, and should be discontinued if it increases distress, dependence, or sound intolerance.

#### 5.1.4. Medication and Supplements

No drug or supplement has robust evidence as a general treatment for chronic subjective tinnitus. Pharmacotherapy should target a defined comorbid condition such as depression, anxiety, migraine, insomnia, or Ménière disease, with attention to interactions and potential ototoxicity. Routine prescribing of antidepressants, anticonvulsants, benzodiazepines, or dietary supplements solely for tinnitus is not supported by major guidelines [9,12,13,14,15]. This distinction protects patients from prolonged treatment without a clear target.

### 5.2. Dental and Temporomandibular Management

The dental objective is to diagnose and manage TMD or orofacial pain, then observe whether tinnitus changes alongside the treated target. Association does not establish causation: systematic reviews find that TMD and tinnitus commonly co-occur, but case definitions, referral populations, and control groups vary [20,33]. The possibility of a bidirectional relationship, shared risk factors, or symptom amplification should remain open.

First-line management should be conservative and reversible. Depending on the diagnosis, this may include education, reduced parafunctional loading, graded jaw use, self-management, sleep and stress support, physical therapy, and a stabilization appliance when clinically justified. Irreversible occlusal adjustment, extensive restorative alteration, orthodontic repositioning, or surgery should not be undertaken solely to treat tinnitus. Contemporary TMD guidance favors education and supported self-management, mobilization or manual therapy where appropriate, and other non-invasive approaches for chronic TMD pain [34].

Intervention evidence is encouraging but not definitive. A randomized trial in patients with TMD-associated somatic tinnitus reported better outcomes when cervico-mandibular manual therapy was added to exercise and education [35]. A delayed-start randomized study found that multidisciplinary non-invasive orofacial treatment reduced TFI scores in a selected temporomandibular somatosensory subgroup [36]. Systematic review evidence, however, includes few trials across heterogeneous interventions such as splints, physical therapy, and laser therapy [37]. Results support a time-limited therapeutic trial in a well-characterized phenotype, not dental treatment for tinnitus in the absence of TMD.

### 5.3. Physiotherapeutic and Cervical Rehabilitation

Physiotherapy should address demonstrable cervical or craniomandibular impairment and function. Manual or passive techniques may be used selectively when supported by examination findings, but no specific manual technique or intensive manual package has been established as a tinnitus treatment. Active rehabilitation—education, graded movement, mobility, exercise, strengthening, endurance, task exposure, and self-management—can be added according to functional deficits and patient goals. Manipulative techniques require appropriate clinician competence, informed consent, and screening for neurologic, vascular, and other contraindications.

The evidence base remains small and heterogeneous. A systematic review suggested possible benefit of physical therapy in selected patients but highlighted limited study quality and inconsistent diagnostic criteria [38]. A delayed-start randomized trial reported improvement in tinnitus and neck-related outcomes after multimodal cervical physical therapy [39]. Other randomized studies have reported benefits from exercise-containing programs or short physical therapy courses [40,41], and a 2025 pilot trial reported improvement after manual therapy plus stretching and relaxation [42]. These studies do not establish a universal cervical treatment for tinnitus or superiority of any manual, manipulative, mobilization, soft-tissue, or active technique.

A practical treatment trial should document the baseline cervical and craniomandibular targets, specify the treatment components and dose, define tinnitus and functional outcomes, record adverse events, and set adaptation and stop points. Any protocol-specific intensive manual strategy should be regarded as investigational until comparative data are available. Lack of improvement in predefined targets should prompt reassessment rather than indefinite escalation. New neurologic or vascular features require medical review.

### 5.4. Behavioral, Sleep, and Participation Support as Complementary Domains

CBT has the most consistent evidence for reducing the negative impact of tinnitus. It targets threat interpretation, attention, avoidance, safety behaviors, and coping rather than promising to erase the sound. A Cochrane review found low- to moderate-certainty evidence for improved tinnitus-related quality of life at treatment end [43]. Specialized stepped CBT-based care outperformed usual care in a large randomized trial, and network meta-analysis supports cognitive and behavioral approaches despite heterogeneity in delivery [44,45]. Guided internet-delivered CBT can improve access and has shown benefit compared with monitoring or non-specialized care [46].

Insomnia deserves direct treatment. In a randomized trial of people with tinnitus-related insomnia, CBT for insomnia improved sleep and also reduced tinnitus distress [47]. Mindfulness-based cognitive therapy may help selected patients, although it should be presented as a way to alter reactivity and functioning, not as a guaranteed percept-reduction intervention [48]. Mental health referral is urgent when suicide risk is present and appropriate when severe depression, anxiety, trauma-related symptoms, or other conditions exceed the scope of routine tinnitus counseling.

Behavioral and sleep care should not be used to dismiss plausible auditory or somatosensory contributors. Conversely, the presence of a jaw or neck phenotype should not postpone evidence-based treatment of disabling insomnia or catastrophic beliefs. The integrated formulation assigns each intervention a target and permits domains to be treated in parallel when clinically necessary. The evidence positions and safeguards for the principal management components are summarized in Table 2.

## 6. Clinical Integration of Otolaryngological, Dental, and Physiotherapeutic Care

### 6.1. The Integration Architecture

A multi-integrated pathway can be implemented with five commitments: one shared baseline, one problem list and formulation, phenotype-gated care modules, one common outcome set, and one scheduled interdisciplinary review. The pathway does not require co-location, simultaneous appointments, or treatment by every discipline. Many tinnitus clinicians already coordinate care across disciplines; the proposal here is not that integration itself is novel. The contribution is to make the organizational logic explicit enough to be audited and prospectively tested.

The coordinator may be an audiologist, otolaryngologist, primary-care clinician, rehabilitation physician, or another appropriately trained professional depending on local resources. Coordination is a function, not a professional monopoly. The coordinator confirms that red flags were addressed, maintains the shared formulation, prevents contradictory explanations, monitors overall treatment burden, and triggers review when progress stalls.

### 6.2. Proposed Phenotype-Gated Integrated Clinical Framework

The proposed phenotype-gated integrated clinical framework links otolaryngological and audiological assessment, dental and temporomandibular care, and physiotherapeutic rehabilitation. It is presented neutrally and is separate from any specific device or experimental technology. Diagnostic triage, audiometry, conservative TMD management, selected cervical or craniomandibular rehabilitation, education, and shared follow-up retain the evidence base of their individual components; they do not acquire additional evidence simply by being placed within the framework.

No peer-reviewed comparative study was identified that validates the complete pathway as a treatment package. The present framework should be regarded as a hypothesis-generating organizational model rather than a validated treatment package, and future prospective studies should compare it with usual or standard care using both clinical and health-economic outcomes.

### 6.3. A Staged Clinical Pathway

At stage 1, the team establishes safety, hearing status, tinnitus burden, functional goals, and urgent referrals. At stage 2, it maps candidate auditory, TMD/orofacial, cervical, sleep, psychological, and contextual phenotypes. At stage 3, the coordinator creates a short formulation distinguishing confirmed diagnoses, plausible contributors, and uncertainty. At stage 4, the patient and team select the smallest set of interventions that addresses the highest-priority targets. At stage 5, all disciplines use a common tinnitus outcome plus domain-specific measures. At stage 6, a planned review decides whether to continue, adapt, de-escalate, stop, or investigate further.

Sequencing should be individualized. Sudden hearing loss or other urgent pathology takes precedence. Communication-limiting hearing loss may be addressed early because improved access to sound and speech can reduce listening effort across other therapies. Severe insomnia or mental health risk may need immediate parallel treatment. A painful TMD or cervical disorder can be treated on its own merits while tinnitus is monitored. Starting multiple low-certainty interventions at once should be avoided when it prevents learning which target changed.

### 6.4. Shared Measurement and Review

A common outcome set should be parsimonious. The TFI or THI can serve as the shared tinnitus measure, but the team must add outcomes that match active targets: hearing and communication goals for audiology; pain and jaw function for TMD; neck function and activity tolerance for physiotherapy; insomnia severity for sleep care; and mood or anxiety measures when clinically indicated. Patient-defined goal attainment, adverse effects, adherence, and treatment burden should be reviewed alongside scale scores.

A review interval of approximately 6 to 12 weeks is often practical for conservative rehabilitation, but timing should reflect the intervention and clinical risk. Predefined adaptation rules are more important than a universal calendar. For example, an unused hearing aid requires fitting and goal review; persistent jaw pain may require diagnostic reconsideration; physiotherapy without functional change should prompt reassessment of its manual and active components; and CBT without engagement may need a different format or intensity. The proposed workflow is summarized in Figure 1, and the corresponding stage-specific responsibilities and minimum outputs are detailed in Table 3.

## 7. Clinical Implications: Precision Without Overdiagnosis

Phenotype-guided care is not equivalent to assigning a cause to every symptom. Modulation by movement, a tender muscle, or an imaging finding can be clinically informative without proving causation. The safest language is probabilistic: a finding is compatible with a somatosensory contribution; a time-limited intervention can test whether the target and tinnitus co-improve; and non-response should lower confidence in the working formulation.

Integration should reduce rather than amplify treatment burden. Each component needs an indication, target, expected time course, outcome, and stop rule. This prevents a patient with bilateral hearing loss and insomnia from being sent automatically for occlusal treatment, or a patient with painful TMD from receiving only generic sound therapy. It also discourages the accumulation of supplements, devices, and passive procedures that cannot be evaluated separately.

Shared language is essential. Teams should avoid competing explanations such as ‘the ear is normal, so the problem is psychological’ or ‘a neck finding proves the tinnitus is cervical.’ A written formulation can state that auditory deafferentation may contribute to the percept, TMD or cervical dysfunction may modulate it, sleep and threat appraisal may amplify burden, and uncertainty remains. This model validates the patient’s experience while preserving scientific caution.

Equity and feasibility matter. A pathway that requires multiple private specialists may widen access gaps. Screening and coordination can be concentrated in one service, with telehealth, digital CBT, community physiotherapy, dental referral only when indicated, and stepped intensity. Implementation outcomes should include waiting time, cost, treatment burden, adherence, and reach, not only symptom change.

## 8. Future Perspectives

### 8.1. Better Phenotyping and Patient Selection

Future studies should move beyond binary labels such as somatic versus non-somatic tinnitus. Auditory, TMD/orofacial, cervical, sleep, psychological, hyperacusis, and participation domains can coexist and vary over time. Repeated phenotyping should test whether temporal co-variation and treatment response improve classification beyond single maneuvers. Biomarkers may become useful, but near-term progress is more likely from standardized clinical definitions, reliable measurement, and transparent eligibility criteria.

### 8.2. Evaluation of Complete Care Pathways

Most available trials evaluate individual components or multi-component interventions in narrow phenotypes. The clinical–organizational model itself should now become a research subject. Pragmatic prospective studies should compare coordinated multi-integrated care with usual or standard care while preserving phenotype-gated treatment. They should evaluate clinical outcomes together with implementation and health-economic outcomes, and should record fidelity, referral activation, sequencing, contamination, treatment burden, harms, and which modules were activated and why. A universal package arm would test a different and less clinically relevant question.

### 8.3. Shared Outcomes and Longitudinal Monitoring

Consensus on a compact outcome set would improve comparison across disciplines. Tinnitus burden should be paired with the outcome targeted by each active module, plus patient-defined goals and adverse effects. Psychoacoustic change should not substitute for functional benefit. Recent work shows that commonly used tinnitus questionnaires capture overlapping but non-identical symptom sets, reinforcing the need to preselect instruments and interpret change in context [49]. Longer follow-up is needed to distinguish transient modulation from durable gains in sleep, communication, activity, and participation.

### 8.4. Digital Monitoring and Artificial Intelligence

Digital diaries and ecological momentary assessment can capture within-person variation in tinnitus, sound exposure, sleep, jaw or neck symptoms, and context more accurately than distant recall. European tinnitus research has demonstrated the feasibility of mobile ecological momentary assessment infrastructure [50]. These tools could support coordinated review by showing whether domains co-vary and whether a specific intervention changes its intended target.

Artificial intelligence may eventually assist pattern recognition, triage, and prediction, but models will reproduce the limitations of their training data. Development should prioritize explainability, prospective validation, privacy, missing-data handling, and performance across age, language, hearing, and digital-literacy groups. Algorithms should support shared decisions rather than assign causal phenotypes or treatment packages autonomously.

### 8.5. Emerging Therapeutic Technologies

Neuromodulation and combination technologies remain research areas rather than components of the proposed care framework. Systematic reviews report modality-specific signals but substantial heterogeneity in targets, dosing, controls, outcomes, and follow-up [51,52]. Studies of sound paired with tongue stimulation show feasibility and symptom improvement, but their designs do not justify generalization to all bimodal systems [53,54]. Personalized sound combined with electromagnetic stimulation has also been explored in an uncontrolled cohort [55]. These technologies require modality-specific evidence, appropriate comparators, safety monitoring, clinically meaningful outcomes, and independent replication before routine integration into care.

### 8.6. Implementation Science and Value

Future studies should test whether coordinated care changes how healthcare is used, not only how symptoms change. Prespecified organizational endpoints should include the number of consultations and specialist referrals, duplicated investigations or procedures, waiting time, treatment burden, adherence, and overall healthcare resource utilization. Economic analyses should capture direct medical costs, patient and caregiver time, travel and productivity losses, and should evaluate cost-effectiveness; where appropriate, quality-adjusted life-years (QALYs) and broader healthcare burden can also be included. These outcomes should be assessed alongside tinnitus burden, function, adverse events, and patient-defined goals.

## 9. Limitations

This is a narrative rather than systematic review. Although the search strategy and evidence hierarchy were explicit, study selection was interpretive, formal duplicate screening and risk-of-bias scoring were not performed, and publication bias cannot be excluded. The literature is heterogeneous in tinnitus definitions, phenotype criteria, interventions, comparators, and outcome instruments. Dental and physiotherapeutic trials are generally small and restricted to selected samples, and associations between TMD, cervical dysfunction, and tinnitus do not establish causality.

Few studies evaluate a truly coordinated interdisciplinary pathway, making it difficult to separate the effects of patient selection, attention, sequencing, individual components, and team communication. The proposed phenotype-gated integrated framework has not been validated as a complete package in a peer-reviewed comparative study. Claims about its clinical effectiveness, efficiency, or cost-effectiveness are therefore hypotheses that require prospective testing. Digital and emerging technological approaches also evolve rapidly and are included here as research directions rather than established additions to routine care.

## 10. Conclusions

Tinnitus should be approached as a heterogeneous percept and, when disabling, as a disorder shaped by auditory, somatosensory, sleep, psychological, and contextual domains. Otolaryngological and audiological assessment provides the diagnostic and hearing foundation. Dental and temporomandibular care and cervical physiotherapy may address clinically relevant somatosensory contributors in selected patients, using conservative, reversible, and goal-based treatment. CBT-based care and insomnia treatment remain important complementary domains with comparatively stronger evidence for reducing burden.

The central organizing idea of the proposed multi-integrated approach is coordination: one baseline, one shared formulation, phenotype-gated modules, common outcomes, a named coordinator, and planned reassessment. This framework is not a validated treatment package and should not be interpreted as superior to existing integrated care or usual care. Its value is as a hypothesis-generating clinical–organizational model whose clinical effectiveness, implementation burden, and cost-effectiveness require prospective comparison with usual or standard care.

## Figures and Tables

**Figure 1:**
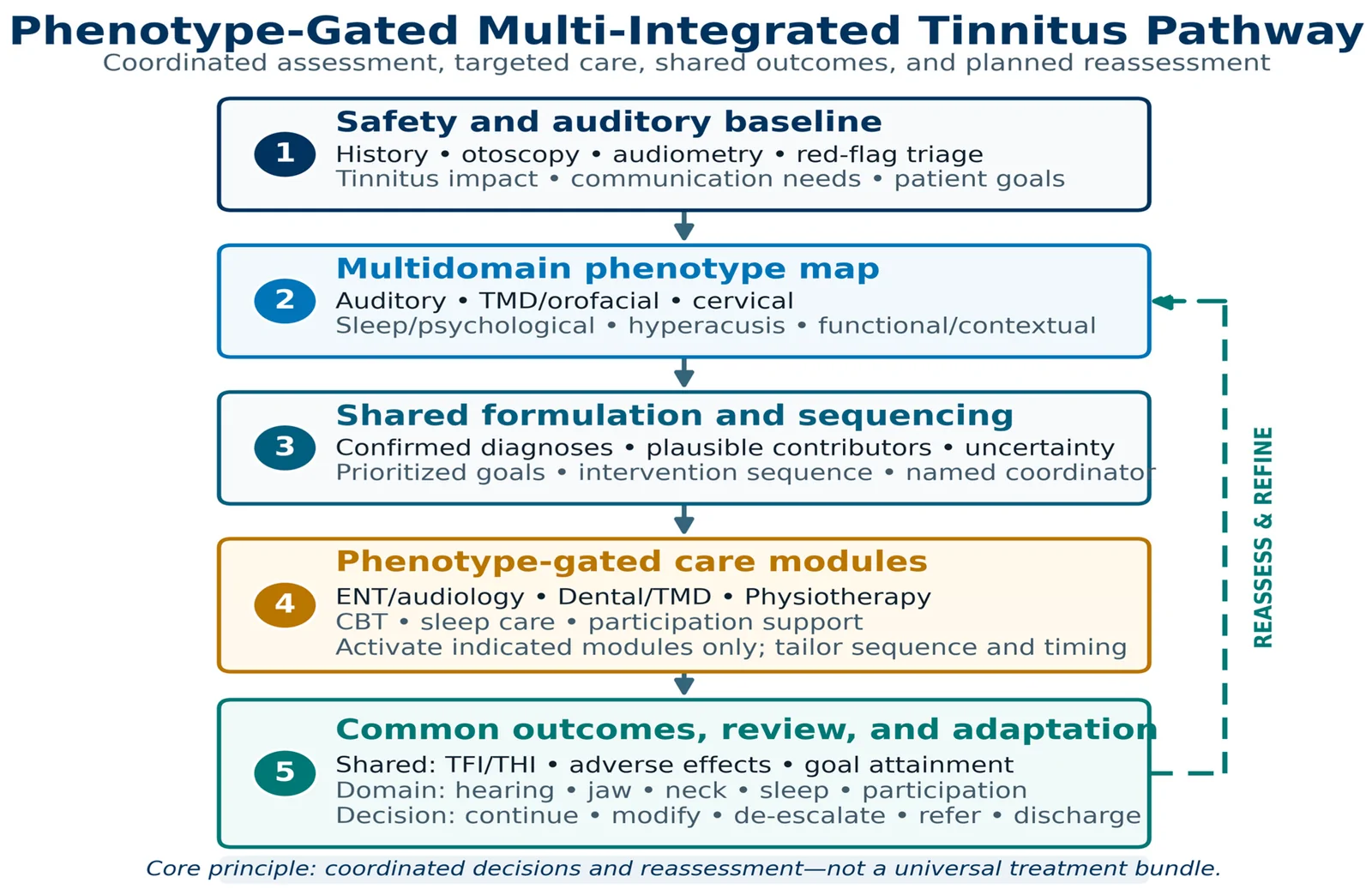
Proposed phenotype-gated multi-integrated tinnitus framework. Integration is defined by shared formulation, targeted modules, common outcomes, and feedback; it does not mean that every patient receives every intervention. The figure represents a hypothesis-generating organizational model, not a validated treatment package.

**Table 1:** Components of a phenotype-guided multi-integrated tinnitus assessment.

Clinical Domain	Assessment Focus	Candidate Phenotype	Primary Role	Shared Outcomes
ENT/audiology	History, otoscopy, audiometry, red flags, hearing and communication; objective middle-ear testing when symptoms suggest conductive disease or ETD	Auditory/otologic; hearing-related participation; objectively confirmed middle-ear/ETD differential	Safety, diagnosis, education, hearing rehabilitation, and condition-specific referral	TFI/THI; hearing goals; adverse events
Dental/TMD	DC/TMD-informed history and examination; jaw pain/function; temporal co-variation	TMD-associated orofacial or somatosensory features	Diagnose and conservatively manage TMD; test whether target and tinnitus co-improve	Jaw pain/function; TFI/THI; goal attainment
Physiotherapy	Neck pain, movement, strength/endurance, load tolerance, trauma, symptom co-variation	Cervical somatosensory phenotype	Phenotype-gated rehabilitation for demonstrable cervical/craniomandibular impairment; use manual techniques selectively and integrate active care as indicated	Neck function; TFI/THI; activity tolerance
Behavioral/sleep	Distress, threat beliefs, avoidance, insomnia, mood, safety, coping	Tinnitus disorder with distress and/or sleep impairment	CBT-based care, CBT-I, risk management, participation support	TFI/THI; sleep; mood; valued activity
Coordinated review	One problem list, sequencing, adherence, burden, conflicting explanations	Mixed or complex phenotype	Integrate decisions, de-escalate ineffective care, and revise formulation	Common outcome set; patient-defined goals; care burden

**Table 2:** Evidence-positioned management components and safeguards.

Component	Best Candidate	Evidence Position	Safeguard or Stop Rule
Education and shared decisions	All patients seeking care	Tier 1: consistently guideline-supported	Revise the explanation if fear, conflicting messages, or unrealistic cure expectations persist
Hearing aids/communication rehabilitation	Communication-relevant hearing loss	Tier 1: guideline-supported for hearing loss; tinnitus-specific outcomes remain variable	Verify fitting and hearing goals; do not prescribe solely on an untested tinnitus claim
Sound enrichment	Patients who find sound useful for coping, contrast, or sleep	Guideline-recognized option; low-certainty comparative evidence	Stop if aversive, ineffective, unsafe, or reinforcing dependence
CBT-based care	Bothersome tinnitus, distress, avoidance, maladaptive beliefs	Tier 1: strongest intervention evidence for reducing tinnitus-related burden	Change intensity or format when engagement or goal movement is absent
CBT for insomnia	Clinically significant tinnitus-related insomnia	Evidence-supported for clinically significant insomnia; focused tinnitus randomized trial	Reassess adherence and alternative sleep disorders if insomnia persists
Dental/TMD care	DC/TMD-compatible findings plus a credible temporal or modulatory relationship	Tier 2: phenotype-specific; promising but low-certainty evidence	Keep treatment conservative and reversible; avoid occlusal escalation for tinnitus alone
Cervical physiotherapy	Neck dysfunction with credible symptom co-variation	Tier 2: phenotype-specific; small heterogeneous trials with low certainty	No manual or active technique has established superiority; screen contraindications, obtain consent, monitor outcomes and adverse events, and modify or stop without meaningful benefit
Neuromodulation or combination technology	Research setting or regulated indication with informed uncertainty	Experimental/research evidence; heterogeneous and modality-specific	Predefine meaningful change, adverse-event monitoring, comparator, and futility criteria

**Table 3:** Operational sequence and minimum outputs for coordinated care.

Stage	Team Action	Lead Responsibility	Minimum Output
1. Safety and baseline	Triage red flags; document hearing, burden, goals, and comorbidities	ENT/audiology or qualified first-contact clinician	Urgency decision, audiological baseline, shared tinnitus measure
2. Phenotype map	Screen auditory, TMD/orofacial, cervical, sleep, psychological, and participation domains	Coordinator with targeted referrals	Reasons for referral; domains not indicated remain inactive
3. Shared formulation	Separate confirmed diagnoses, plausible contributors, and uncertainty	Coordinator plus patient	One written problem list and prioritized goals
4. Targeted care	Activate the smallest clinically justified set of modules	Relevant discipline	Intervention target, dose, expected outcome, safety plan
5. Common measures	Repeat tinnitus and domain-specific outcomes; record adverse effects and burden	Each discipline returns data to coordinator	Comparable follow-up data and patient goal status
6. Team review	Continue, adapt, de-escalate, stop, or investigate further	Coordinator and relevant team members	Updated plan, ownership, next review or discharge criteria

## Data Availability

No new data were created or analyzed in this study. Data sharing is not applicable to this article.

## Supplementary Materials

The following supporting information can be downloaded at: https://www.mdpi.com/article/10.3390/life16101588/s1, Table S1: Main search strings; Table S2: Study-selection and evidence-mapping approach.
