# Supplementary material for: A Multi-Integrated Approach to Tinnitus Phenotype-Guided Otolaryngological, Dental, and Physiotherapeutic Care
Source: Life (Basel). 2026 Sep 23;16(10):1588. doi: 10.3390/life16101588 (PMC13705818; doi:10.3390/life16101588)
Supplement: Supplementary file 1 [file life-16-01588-s001.zip › life-4549948-supplementary.pdf]

SUPPLEMENTARY MATERIALS

A Multi-Integrated Approach to Tinnitus

Phenotype-Guided Otolaryngological, Dental, and Physiotherapeutic Care

Table S1. Main search strings

Search syntax was adapted to the indexing and interface of each source; the strings below report the principal concepts and Boolean structure.

Source	Main search string or approach	Limits
PubMed/MEDLINE—integrated tinnitus domains	("Tinnitus"[Mesh] OR tinnitus[tiab]) AND (audiolog*[tiab] OR "Hearing Loss"[Mesh] OR "Hearing Aids"[Mesh] OR "Cochlear Implants"[Mesh] OR "sound therapy"[tiab] OR somatosensory[tiab] OR somatic[tiab] OR "Temporomandibular Joint Disorders"[Mesh] OR bruxism[tiab] OR orofacial[tiab] OR cervical[tiab] OR physiotherap*[tiab] OR "physical therapy"[tiab] OR rehabilitation[tiab] OR "Cognitive Behavioral Therapy"[Mesh] OR sleep[tiab] OR insomnia[tiab] OR multidisciplinary[tiab] OR integrat*[tiab] OR "ecological momentary assessment"[tiab] OR neuromodulation[tiab])	English; 1 January 2014–12 August 2026. Seminal earlier sources were retained when they defined criteria, outcomes, mechanisms, or enduring guidance.
PubMed/MEDLINE—nasal, Eustachian-tube, and oxygenation concepts	(tinnitus[tiab] OR "Tinnitus"[Mesh]) AND ("Eustachian tube dysfunction"[tiab] OR "Eustachian tube stenosis"[tiab] OR "balloon dilation tuboplasty"[tiab] OR "nasal obstruction"[tiab] OR "middle-ear ventilation"[tiab] OR hypoventilation[tiab] OR hypoxi*[tiab] OR "sleep apnea"[tiab])	English; no date restriction when locating the two prespecified Di Rienzo Businco Eustachian-tube studies and relevant consensus work.
Cochrane Library	tinnitus AND (hearing aid* OR sound therapy OR cognitive behavio?r* therapy OR sleep OR insomnia OR temporomandibular OR cervical OR physiotherapy OR neuromodulation)	Reviews and controlled trials relevant to adult subjective tinnitus.
Guidelines and citation chaining	Targeted searches of NICE, VA/DoD, AAO-HNSF, the German S3 program, and European tinnitus guidance; backward and forward citation searches from guidelines, systematic reviews, pivotal trials, and newly included ENT sources.	Sources were retained when directly relevant to assessment, phenotype selection, intervention evidence, outcomes, safety, or care-pathway integration.

Table S2. Study-selection and evidence-mapping approach

Element	Operational approach
Scope	Adult subjective tinnitus, with emphasis on non-pulsatile tinnitus and on auditory/otologic, dental/TMD, cervical/physiotherapeutic, sleep, behavioral, and care-integration domains.
Eligibility	English-language guidelines, systematic reviews, randomized or prospective comparative studies, and clinically informative observational or mechanistic studies that addressed the review aims. Seminal earlier sources were eligible when they defined a diagnostic construct, outcome instrument, mechanism, or enduring recommendation.
Exclusions	Duplicate reports; sources outside the stated clinical scope; papers lacking a relevant tinnitus, phenotype, assessment, intervention, safety, outcome, or service-delivery contribution; and promotional or technical descriptions without peer-reviewed clinical evidence, except where transparently identified as investigational context.
Screening process	Potentially relevant titles and abstracts were assessed for topical fit; full texts were then evaluated for design, population, directness, and clinical applicability. Selection was not performed independently in duplicate, and a PRISMA flow count was not generated because this was a structured narrative review.
Evidence hierarchy	Guidelines, systematic reviews, randomized trials, and prospective comparative studies were prioritized. Observational evidence was used for association, prevalence, feasibility, or phenotype description and was not interpreted as proof of causation or efficacy.
Synthesis and stopping rule	Each retained source was mapped to a prespecified thematic domain and interpreted according to design, consistency, directness, and applicability. Searches ended on the fixed final-search date of 12 August 2026; no subjective or formal saturation threshold was used.
Updated ENT evidence	The two prespecified Di Rienzo Businco Eustachian-tube studies were included as uncontrolled clinical series. They inform ENT phenotyping and tubal-function assessment but were not treated as comparative evidence that an intervention improves tinnitus.
